# Quantitative evaluation of variations in rule-based classifications of land cover in urban neighbourhoods using WorldView-2 imagery

**DOI:** 10.1016/j.isprsjprs.2013.11.007

**Published:** 2014-01

**Authors:** Mariana Belgiu, Lucian Drǎguţ, Josef Strobl

**Affiliations:** a Department of Geoinformatics (Z_GIS), Salzburg University, Schillerstr. 30, 5020 Salzburg, Austria; b West University of Timisoara, Department of Geography, Vasile Parvan Avenue, 300223 Timisoara, Romania

**Keywords:** Land Cover, Comparison, Image, Accuracy, Urban, Experiment, OBIA

## Abstract

The increasing availability of high resolution imagery has triggered the need for automated image analysis techniques, with reduced human intervention and reproducible analysis procedures. The knowledge gained in the past might be of use to achieving this goal, if systematically organized into libraries which would guide the image analysis procedure. In this study we aimed at evaluating the variability of digital classifications carried out by three experts who were all assigned the same interpretation task. Besides the three classifications performed by independent operators, we developed an additional rule-based classification that relied on the image classifications best practices found in the literature, and used it as a surrogate for libraries of object characteristics. The results showed statistically significant differences among all operators who classified the same reference imagery. The classifications carried out by the experts achieved satisfactory results when transferred to another area for extracting the same classes of interest, without modification of the developed rules.

## 1 Introduction

Very High Resolution (VHR) sensors such as IKONOS, OrbView, QuickBird and WorldView-2 (WV2) (DigitalGlobe, Inc., USA) allow accurate mapping of land cover classes in urban/suburban neighbourhoods (Carleer and Wolff, 2006, Herold et al., 2003, Im et al., 2012, Kumar et al., 2012, Pacifici et al., 2009). However, mapping land cover classes in a timely and accurate manner is challenged by the high within-class spectral variation and the spectral similarity between different classes (Goetz et al., 2003, Lu et al., 2011, Myint et al., 2011, Slonecker et al., 2001, Zhou and Troy, 2008). These problems cannot be solved by the traditional per-pixel approach, using only spectral information in the image classification procedure (Benz et al., 2004, Blaschke and Strobl, 2001, Yu et al., 2006). Therefore, new methods are required to solve the challenges triggered by VHR.

In the last years, the Object Based-Image Analysis (OBIA) method has been accepted as an efficient method to classify high-resolution imagery (Blaschke, 2010). OBIA is an iterative image analysis method starting with the partition of the image into homogeneous image objects, through image segmentation (Baatz and Schäpe, 2000). The resulting objects are used as input for the subsequent classification task, whose results are visually inspected and refined if necessary (Benz et al., 2004).

The accuracy and reliability of the OBIA approach depend to a large extent on the image segmentation method and strategy (Baatz and Schäpe, 2000, Benz et al., 2004). The available segmentation algorithms control the segmentation outputs by certain parameters (Liu et al., 2012). For instance, in the Multi-Resolution Segmentation (MRS) algorithm (Baatz and Schäpe, 2000), the scale parameter (SP), colour and shape are user-defined parameters. Since the selection of the optimal segmentation parameters is often a trial-and-error procedure (Hay and Castilla, 2008), new methods were proposed for their objective identification, based on quantitative approaches (Anders et al., 2011, Drǎguţ et al., 2010).

Once the image objects are generated, a large number of object characteristics (referred to as image object features) can be computed and used in the subsequent classification task such as: multi-spectral information (brightness, ratios, standard deviation), shape characteristics, and spatial and hierarchical relations (Benz et al., 2004, Hu and Weng, 2010). There are two types of classification procedures for assigning image objects to the desired land cover classes namely rule-based classification, aka membership function classifier (providing fuzzy or crisp membership functions), and the Nearest Neighbour classifier (Myint et al., 2011). The latter assigns the image objects to the classes of interest according to their similarity to selected training samples and defined feature space (indices, texture, spectral information etc.). The dependence of this classification technique upon the training samples makes it less transferable to other images (Hodgson et al., 2003). Rule-based classification on the other hand relies on the a priori knowledge that can be re-used to classify the desired geographic objects. This classification procedure is rapidly gaining in importance as it allows the image analysts to evaluate in detail and transparently the characteristics of the image objects, as well as the spectral similarities and differences between them, when defining the class membership conditions (Baltsavias, 2004, Xu, 2013). Nevertheless, building the rules is not a trivial task. The large number of the available image features greatly challenges the image operators who have to determine the most relevant features and corresponding thresholds to classify the image objects. Three solutions have been identified to define the rules: by means of automatic induction methods (data mining methods), using cognitive methods, or by “explicitly eliciting the rules from the experts” (Hodgson et al., 2003). Previous research has used data mining techniques to select the optimal features for the rule-based classifications (Carleer and Wolff, 2006, de Pinho et al., 2012, Tullis and Jensen, 2003). However, this procedure is empirically tuned to the analyzed data and is hardly transferable to other areas. Other studies have developed classification rules based on human knowledge acquired by interviewing the domain experts (Kohli et al., 2012), by mimicking photo-interpreters knowledge (Lloyd et al., 2002, Sebari and He, 2013), or by using expert knowledge gained through praxis (Myint et al., 2011).

In photointerpretation, the operators work with libraries (visual interpretation keys) of “known spatial, texture and colour patterns” (Zhou et al., 2010). Unfortunately, OBIA lacks libraries of image object characteristics that might contribute to the development and optimization of automatic and more transferable rulesets (Eisank et al., 2011, Anders et al., 2011; Arvor et al., 2013). Furthermore, the considerable time spent on developing the rulesets, besides the identification of the relevant segmentation parameters, seriously impedes the application of OBIA in operational frameworks (Baker et al., 2013, Duro et al., 2012), where “the speed and flexibility with which information is produced is an important factor” (Moller-Jensen, 1997). In the absence of a systematic approach to conceptualize and formalize the classification through rulesets, OBIA remains a subjective, error-prone and hardly reproducible method (Arvor et al., 2013).

There are many examples of studies which evaluate how different operators conceptualize and delineate manually the features of interest from the data at hand (Albrecht et al., 2010, Ardelean et al., 2013, Corcoran et al., 2010, Edwards and Lowell, 1996, Zhou et al., 2010). Gardin et al. (2010) emphasized the need for methods to evaluate the nature of errors that might affect the digitizing of remote sensing images. They used a web-based framework to collect the demographic characteristics, visual working memory and psychological personality profile that might influence the operator performance on remote sensing image interpretation tasks (Gardin et al., 2010). These studies showed that extraction of objects from raw data is biased by human subjectivity, which leads to differences in the results produced by various operators. Such differences are expected in object-based classifications as well. However, a systematic quantification of the variability of the rule-based classifications carried out by independent operators is missing in OBIA.

The objective of this paper is to assess the variability in the results of OBIA rule-based image classifications carried out by different experts who were all assigned the same interpretation task. The magnitude of differences was quantified with kappa statistics, and the statistical significance of the differences between pair-wise classifications was evaluated using the McNemar’s test (Agresti, 1996, Bradley, 1968, Foody, 2004). We developed an additional fuzzy ruleset classification based on the previous studies dedicated to mapping urban/suburban land cover classes. This new classification model serves as an additional classification model to be used in our rulesets variability test. The transferability of the developed classification rulesets has been assessed by applying them to an additional test area that covers 90% of the initial site, but with a much larger extent. Through this test, we aimed at evaluating the degree to which the developed rule-based classifications reach comparable results on an additional image.

This paper is organized as follows: Section 2 introduces the study area, describes the experiment carried out to evaluate the differences (variability) between the digital rule-based classifications carried out by three independent experts (referred to as C1–C3 classifications), and introduces our own methodology used to classify pre-defined land cover classes from the imagery. The next section (Section 3) is dedicated to the results, before the discussions (Section 4) and conclusions of this work (Section 5) are presented.

## 2 Methods and data

### 2.1 Study area and data

**Fig. 1:**
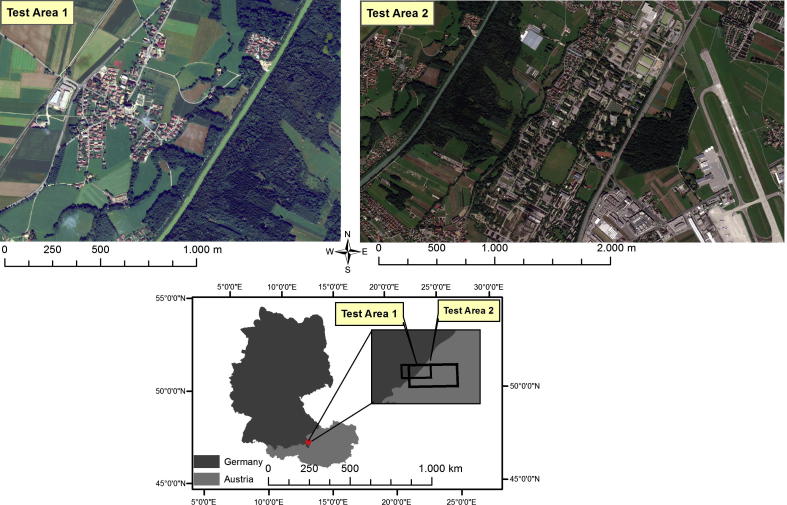
Study Area: Test Area 1; Test Area 2. Colour composite images: red (Band 5), green (Band 3), blue (Band 2).

The study areas are situated at the border between Salzburg, Austria and Bavaria, Germany (Fig. 1). The first test site represents a typical suburban area composed of extended vegetation areas (forest, meadow-like zones, and agricultural fields) and complex human settlements. It has an extent of 3556 × 2521 pixels. The second test area has a larger extent (7328 × 4181 pixels) and covers 90% of the first test site, including an additional industrial area composed of large industrial buildings and dispersed residential houses. The data used in this study was a pan-sharpened WV2 image, acquired on September 11, 2010. WV2 is the first high-resolution eight-band commercial satellite (see Table 1 for more details).

**Table 1:** Characteristics of WorldView-2 imagery.

Spectral bands	Wavelength (nm)	Spatial Resolution (m)
1 Coastal blue	400–450	2
2 Blue	450–510	2
3 Green	510–580	2
4 Yellow	585–625	2
5 Red	630–690	2
6 Red-edge	705–745	2
7 NIR1	770–895	2
8 NIR2	860–1040	2
Pan	450–800	0.5

### 2.2 Methodology

#### 2.2.1 Experimental setting

In this study, we evaluated the image classifications carried out by three image analysts who had to map the following land cover classes: impervious areas, bare soil, vegetation and water areas. To avoid the potential classification variability generated by different conceptualizations of the real-world object semantics, the experts were provided with detailed descriptions of the classes to be identified in the image. We aimed at quantifying the variability induced by the application of different rulesets to extract information from imagery, rather than assessing the differences on the semantic interpretation of real world objects.

The image analysts could define additional classes (e.g. shadow class) that might help them to distinguish between pre-defined classes or to improve the classification accuracy. They had to fill in a form with the following information: experience with OBIA methods, experience with WV2 imagery analysis and the time spent to carry out the classification. All three image analysts have a solid experience with OBIA, but none of them had used WV2 before.

#### 2.2.2 A ruleset based on best practices in literature

**Fig. 2:**
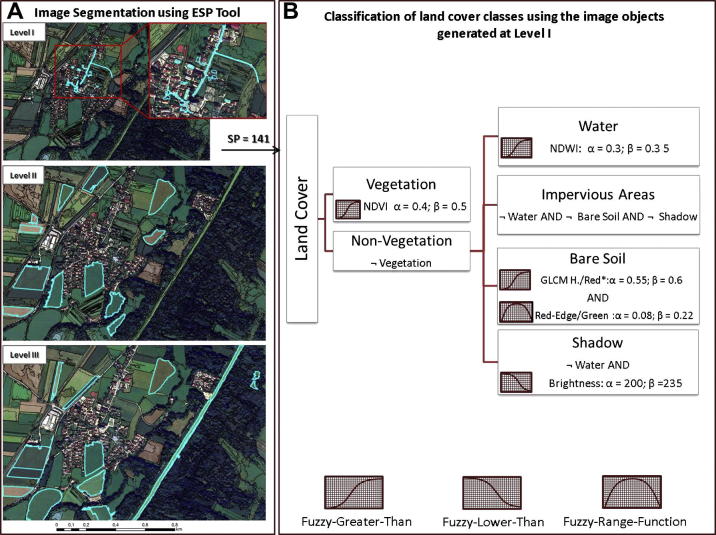
Rule-based classification of selected land covers classes. The displayed segmentation layers (Level I, Level II, Level III) were generated using the ESP tool. The image objects identified as Level I (predicted Scale Parameter (SP) is 141) were used as the input segmentation layer for the subsequent classification tasks. *α* – lower border concerning a property *p*; *β* – indicates the upper border; – complement class (not) ^∗^ Texture GLCM Homogeneity (quick8/11) on Red Band.

We developed an additional rule-based classification model that relies on the common-sense knowledge gained so far in mapping impervious surfaces in urban/suburban environments using VHR imagery. The development strategy, design, and structure of the rule sets followed the approach represented in Fig. 2. Prior to classification, the image was segmented by applying the MRS algorithm (Fig. 2, step A). The MRS requires the definition of different parameters that control the homogeneity of the resulting image objects. To predict proper SPs to delineate the classes of interest, we applied an improved version of the ESP tool (Drǎguţ et al., 2010), which works on multiple layers (Drǎguţ et al., in preparation). The new tool automatically identified patterns in the data at three different scales (Fig. 2), from fine objects (Level I – SP = 141), to larger regions (Level II – SP = 201; Level III – SP = 401), in a data-driven approach. After visually inspecting the segmentation results at all three image segmentation levels, we selected the finest image segmentation scale (Level I), which produced image objects that match the the desired geographic objects (see Fig. 2 for more details). The image objects generated at this level were further used as building blocks in the classification (Fig. 2, step B).

Following the image segmentation step, a two-level classification hierarchy was created: vegetation and non-vegetation areas were defined on the first hierarchy level, whereas the other classes were defined as subclasses of the non-vegetation class. The classification rulesets define the membership of the image objects to a given class by means of fuzzy functions (Fig. 2, step B). Thus, each class was defined by one or more fuzzy membership functions, which were combined by using the fuzzy AND operator. The thresholds of the features used to allocate the image objects to the proper land cover class were set manually.

Vegetation areas were masked out using the Normalized Difference Vegetation Index (NDVI) (Rouse et al., 1973). Previous studies have shown the potential of the additional bands of the WV2 satellite to improve the tree species mapping (Pu and Landry, 2012), and impervious surface extraction (Im et al., 2012). Kumar et al. (2012) tested the potential of the new WV2 imagery bands to discriminate vegetated areas from non-vegetated areas. They proved that vegetation classification performed better by using the NIR2 and Red-Edge bands. We tested these bands too, but this approach did not achieve improved results over the NDVI index calculated with NIR1 band. Therefore, the NDVI was calculated as follows (see Eq. (1)):

(1) $$\mathrm{NDVI}=\frac{\mathrm{NIR}1-\mathrm{Red}}{\mathrm{NIR}1+\mathrm{Red}}$$

The impervious areas class was separated from the soil class based on the GLCM Homogeneity texture (Haralick, 1979) on the Red Band, and an index that we named the RedEdge/Green index (Eq. (2)):

(2) $$\mathrm{RedEdge}/\mathrm{Green}=\frac{\mathrm{RedEdge}-\mathrm{Green}}{\mathrm{RedEdge}+\mathrm{Green}}$$

Water features were classified using the Normalized Difference Water Index (NDWI) (McFeeters, 1996). This index was successfully used to classify and monitor water features (Gao, 1996, Xu, 2006), because it is independent from illumination changes or other image distortions that might lead to inconsistencies in the Digital Number (DN) within the class.

(3) $$\mathrm{NDWI}=\frac{\mathrm{Green}-\mathrm{NIR}1}{\mathrm{Green}+\mathrm{NIR}1}$$

The shadows present in all VHR challenges the image analysis task (Zhou et al., 2009). Existing techniques to solve the shadow problems rely on algorithms for shadow removal or shadow detection and classification as a separate class (Tsai, 2006). We chose the latter approach. Thus, the shadows were separated from other low spectral feature classes (water, dark impervious areas etc.) using the mean brightness (*b*) feature (Zhou and Troy, 2008), calculated as follows (Trimble, 2012):

(4) $$b=\frac{1}{n}+\overset{n}{\underset{j=1}{\sum }}w_{j}b_{i}^{j}$$

where *b* is the ‘brightness’ and *w_j_* ∈ *R*^+^ (*R*-set of real number) and 0 ⩽ *w_j_* ⩽ 1 the weight of channel *j*.

For the sake of simplicity we further refer to our classification as C4 classification.

#### 2.2.3 Accuracy Assessment and classification results comparison

The results of all four classifications were assessed using a standard confusion matrix to calculate the overall accuracy and the Kappa coefficient (Congalton, 1991). The accuracy assessment also includes the producer’s and user’s accuracy in order to evaluate the omission and commission errors for each class (Congalton, 1991). We generated ∼60 samples per class in a stratified random sample scheme, using the results of C4 as strata. The centroids of the image objects were visually interpreted based on the true-colour composite of WV2 (Band 5, Band 3, Band 2), Bing Map Aerial (©2012 Nokia, ©2013 Microsoft Corporation) and Google Maps (GeoBasis-DE/BKG (©2009, Google Map data ©2012). We aimed at a minimum of 50 samples per class (Herold et al., 2008, Scepan, 1999). The differences between classifications were assessed by comparing the kappa coefficient (Congalton, 1991), and by means of *z*-test statistics (Foody, 2004). To evaluate the variability of the classifications, we used the same set of samples. Since the reference data is not independent, the statistical significance of the difference between two classifications was evaluated using the McNemar’s test (Agresti, 1996, Bradley, 1968, Foody, 2004). McNemar’s test is a non-parametric test that is based upon the following formula (Foody, 2004) (see Eq. (5)):

(5) $$z=\frac{f_{12}-f_{21}}{\sqrt{f_{12}+f_{21}}}$$

where *f*_12_ indicates the number of samples correctly classified in the first classification, but incorrectly in Classification2, and *f*_21_ represents the number of samples correctly classified in Classification2, but incorrectly classified in Classification1 (Foody, 2004). The McNemar test has been used in other studies to statistically compare different image classification algorithms (e.g. Duro et al., 2012, Im et al., 2012).

To evaluate the significance of the differences between classifications carried out on Test Area 1 and Test Area 2, the following *z*-test was performed see (Eq. (2)) (Foody, 2004):

(6) $$z=\frac{\frac{x_{1}}{n_{1}}-\frac{x_{2}}{n_{2}}}{\sqrt{p(1-p)\left(\frac{1}{n_{1}}+\frac{1}{n_{2}}\right)}}$$

where *x*_1_ and *x*_2_ represent the number of correctly allocated classes in two independent reference samples of size *n*_1_ and *n*_2_; $p=\frac{x_{1}+x_{2}}{n_{1}+n_{2}}$.

## 3 Results

### 3.1 Experimental results

#### 3.1.1 Differences in the rulesets defined by different experts

**Fig. 3:**
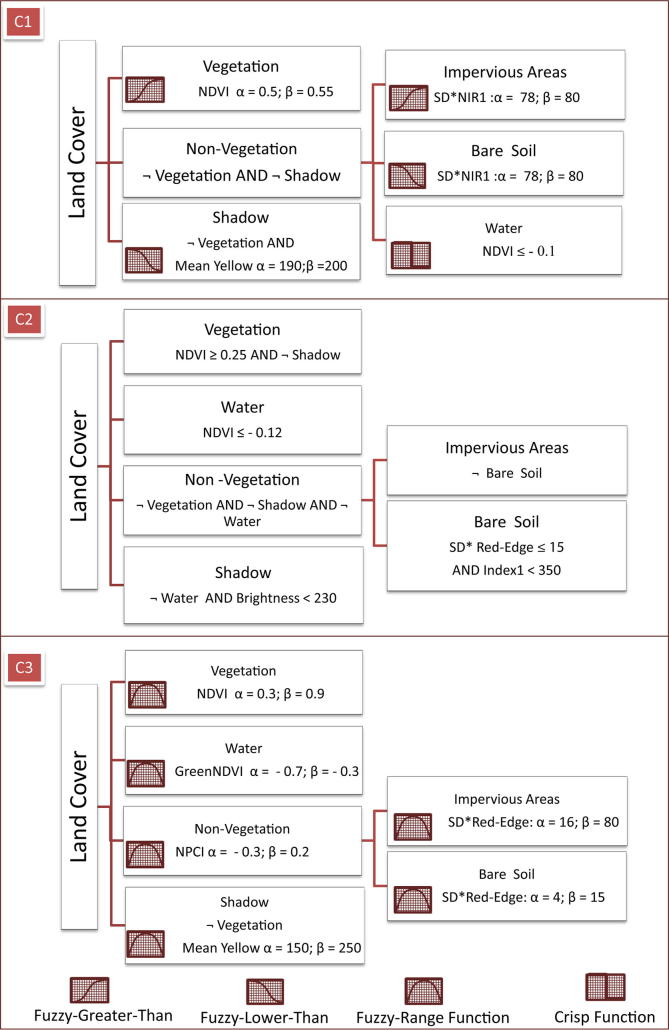
The rulesets defined by the C1–C3 operators; *α* – lower border concerning a property *p*; *β* – indicates the upper border; - complement class (not); ^*^SD – Standard Deviation.

All four image analysts (including ourselves) adopted a hierarchical rule-based classification approach to identify the desired land cover classes from the image (Fig. 2, Fig. 3). However, the image analysis routines differ with respect to the class allocation to the hierarchical levels and the definition of the rulesets. The image analysts spent between 6 and 8 h to carry out the entire classification task.

The image object features used to define the rulesets are explained in Table 2.

**Table 2:** Overview of the image objects features used by C1–C3 operators to classify the pre-defined classes.

Name	Formula	References
Index 1	$\mathrm{Index}1=\frac{\mathrm{Green}+\mathrm{Red}+\mathrm{Blue}}{3}$	
Standard Deviation	Describes the spectral homogeneity of an object. The higher the standard deviation, the less spectrally homogeneous an object is considered Hofmann et al. (2011)	
Normalized pigment chlorophyll index (NPCI)	$\mathrm{NPCI}=\frac{\left[\mathrm{MeanRed}]-[\mathrm{MeanCoastalBlue}\right]}{\left[\mathrm{MeanRed}]+[\mathrm{MeanCoastalBlue}\right]}$	Penuelas et al. (1995); cited by Shamsoddini et al. (2011)
Green NDVI_n_	$\mathrm{GreenNDVI}=\frac{\left[\mathrm{MeanNIR}1]-[\mathrm{MeanGreen}\right]}{\left[\mathrm{MeanNIR}1]+[\mathrm{MeanGreen}\right]}$	Rouse et al. (1973)

The first expert (C1) defined a fuzzy rule-based model to classify the desired land cover classes. The image was segmented using the MRS algorithm (Baatz and Schäpe, 2000) with a SP of 50, shape of 0.1 and compactness of 0.5. The first classification level included shadow, vegetation, and non-vegetation classes. Subsequently, the non-vegetation areas were re-segmented using the MRS with a SP of 300. The newly created image objects were classified as impervious areas, bare soil and water classes. The second expert (C2) applied a Boolean (crisp) rule-based classification approach. The image was segmented with segmentation parameters similar to C1. The first classification level includes shadow, vegetation, water and non-vegetation classes (Fig. 3). No segmentation refinement was performed in this case. The third expert (C3) also defined a fuzzy rule-based model to classify the imagery. The image was segmented using the MRS, the segmentation parameters being similar to C1 and C2. The classification hierarchy levels are similar to C2.

#### 3.1.2 Accuracy assessment results

**Fig. 4:**
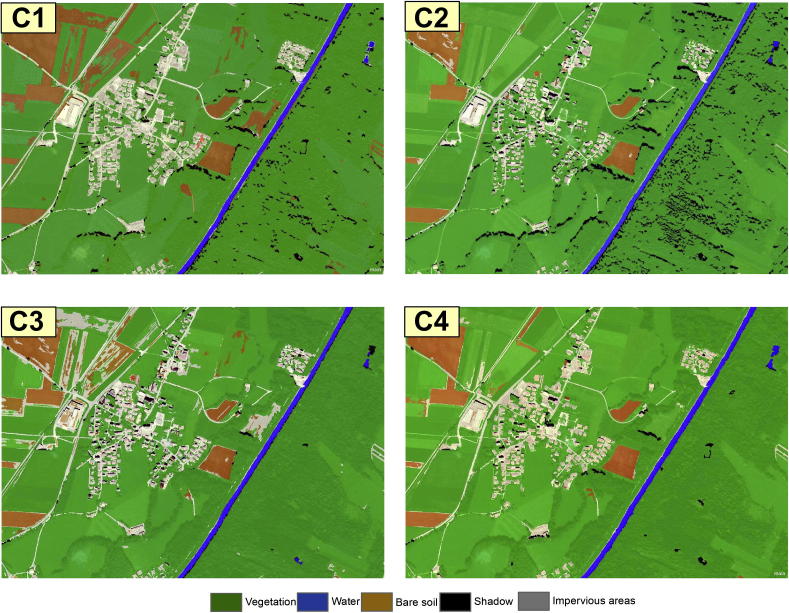
Classification Results Test Area1; C1- expert 1; C2- expert 2; C3- expert 3; C4- classification developed in this paper (author’s classification).

The results of the classifications are depicted in Fig. 4.

**Fig. 5:**
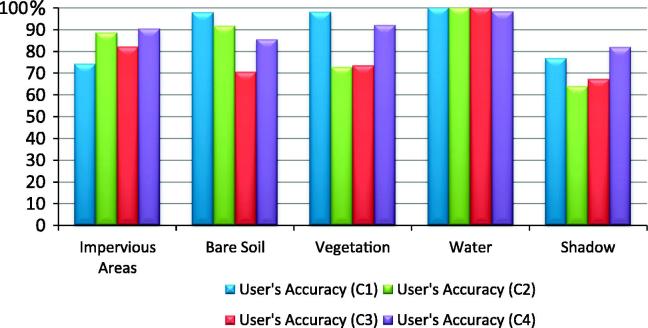
User’s Accuracy per class achieved by C1–C4 classifications in Test Area 1.

**Fig. 6:**
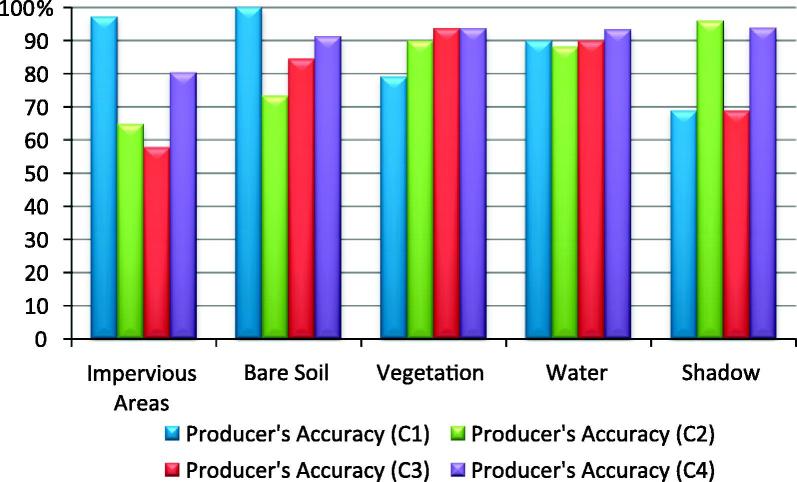
Producer’s Accuracy per class achieved by C1–C4 classifications in Test Area 1.

The ‘Water’ class was the most accurately classified amongst all four analysts (Table 3 and Fig. 5, Fig. 6). The C1–C3 classifications yielded a slightly lower producer’s accuracy value for the water class, because of the confusion with the ‘Shadow’ class (C1 = 89.83%; C2 = 88.14%; C3 = 89.83%). The ‘Vegetation’ class also yielded satisfactory results. All classifications achieved high producer’s accuracy for this class, but the C2 and C3 rulesets yielded lower user’s accuracy (C2-72.60% and C3-73.4%), because of the spectral confusion with the ‘Impervious’ and ‘Shadow’ classes. The ‘Impervious’ class achieved low accuracy in all four classifications (C1–C4). The lowest user’s accuracy for this class was achieved by C1 (74.19%). The commission errors were distributed among all other classes except for the ‘bare soil’. The lowest producer’s accuracies for the impervious areas were achieved by C2 and C3 (64.8% and 57.75% respectively) because of the confusion with the bare soil and the vegetated areas. The C3 achieved lower accuracy because of the overlap between soil and buildings with dark and bright roof. The C2 alleviated the problem of spectral similarity between soil and bright roof buildings by using the Index 1 (see Table 2). Therefore, the C2 rulesets performed slightly better than C3. The C4 yielded an acceptable producer’s accuracy for the ‘Impervious’ class (80.28%). Some impervious areas were misclassified mainly because of the confusion between brown dark roof buildings and bare soil.

**Table 3:** The producer’s and user’s accuracies of the land cover classes achieved by the C1–C4 classifications in Test Area 1; PA%- % producer’s accuracy; UA %- % user’s accuracy; OA – Overall Accuracy (%);kappa (Kappa Index Agreement/Kappa Coefficient); A – Impervious Areas; B – Bare Soil; C – Vegetation; D – Water; E – Shadow.

	C1									C2							
	A	B	C	D	E	Total	PA (%)	UA (%)		A	B	C	D	E	Total	PA (%)	UA (%)
A	69	0	8	2	14	93	97.18	74.19	A	46	4	1	1	0	52	64.79	88.46
B	0	45	0	1	0	46	100.00	97.83	B	3	33	0	0	0	36	73.33	91.67
C	0	0	49	0	1	50	79.03	98.00	C	10	8	53	0	2	73	89.83	72.60
D	0	0	0	53	0	53	89.83	100.00	D	0	0	0	52	0	52	88.14	100.0
E	2	0	5	3	33	43	68.75	76.74	E	12	0	8	6	46	72	95.83	63.89

Total	71	45	62	59	48	285			Total	71	45	62	59	48	285		
							OA	87.36%								OA	80.7%
							kappa	0.84								kappa	0.77
																	
	C3									C4							
A	41	6	3	0	0	50	57.75	82.00	A	57	1	3	0	2	63	80.28	90.48
B	16	38	0	0	0	54	84.44	70.37	B	6	41	0	1	0	48	91.11	85.42
C	4	1	58	1	15	79	93.55	73.42	C	0	3	58	2	0	63	93.55	92.06
D	0	0	0	53	0	53	89.83	100.00	D	0	0	0	55	1	56	93.22	98.21
E	10	0	1	5	33	49	68.75	67.35	E	8	0	1	1	45	55	93.75	81.82

Total	71	45	62	59	48	285			Total	71	45	62	59	48	285		
							OA	78.24%								OA	89.82%
							kappa	0.72								kappa	0.87

The class ‘Bare Soil’ reached low accuracy in all four classifications. The C3 achieved the lowest user’s accuracy (69.8%) for this class because of the spectral confusion with the impervious areas. The ‘Shadow’ class had a low user’s accuracy in all four classifications because of the confusion with impervious areas and the water class (see Discussion section for more details). The lowest producer’s accuracy was achieved by C1 and C3 (68.7% both of them), whereas the lowest user’s accuracy was achieved by C2 (63.89%) because of the confusion with the ‘Impervious’ class.

The experiment showed that the overall accuracy of the classifications varied among the operators (Table 4). C4 yielded an overall accuracy of 89.82% and a kappa coefficient of 0.87, followed by C1 with an overall accuracy of 87.3%, and kappa coefficient of 0.84. C3 achieved a lower overall accuracy (80.7%) and kappa coefficient (0.77), whereas C3 achieved the lowest overall accuracy (78.24%) and kappa coefficient (0.72). The pair-wise McNemar’s tests were performed with the null hypothesis of no significant difference between pairs of distinct classification results (C1 = C2), and the alternative hypothesis of C1 ≠ C2. Variability of the results was found statistically significant in all cases. According to Table 4, the C4 and C1 were significantly different from C2 and C3.

**Table 4:** Summary of the classifications comparison: kappa coefficients comparison and McNemar test pair-wise classifications comparison, alpha: 0.50 (McNemar test relied on Eq. (1)); *k* – kappa coefficient.

		Comparison of overall accuracy		Comparison of kappa coefficients			Comparison of proportions		
Classif.1	Classif.2	Overall accuracy 1 (%)	Overall accuracy 2 (%)	*k*1	*k*2	*k*1–*k*2	|*z*| Observed	|*z*| Critical value	*p* Value
C4	C1	89.82	87.36	0.87	0.84	0.03	2.268	1.960	0.023
C4	C2	89.82	80.7	0.87	0.77	0.10	4.903	1.960	<0.0001
C4	C3	89.82	78.24	0.87	0.72	0.15	5.659	1.960	<0.0001
C1	C2	87.36	80.7	0.84	0.77	0.07	4.129	1.960	<0.,0001
C1	C3	87.36	78.24	0.84	0.72	0.12	5.004	1.960	<0.,0001
C2	C3	80.7	78.24	0.77	0.72	0.05	2.475	1.960	0.013

### 3.2 Testing the performance of developed rulesets on a new test site

To assess the efficiency and robustness of the classification rules, we applied the C1–C4 to a second test area which incorporates 90% of the Test Area 1, but has a larger extent (see Fig. 1, Test Area 2). The same satellite image was used. The difference in the scene extent introduced variations in the size of the image objects generated through segmentation, and therefore we expected differences in the spectral statistics of the image objects. The rule-based classifications were re-used without changing the classification parameters (object features and their thresholds) defined for the first test area. Segmentation was performed with an SP of 50 for the C1–C3 classification procedures, whereas for C4 we used an SP of 161 (as identified by the ESP tool). The classification accuracy was performed using the procedure described for Test Area1 to generate reference sampling data for the new test site.

**Fig. 7:**
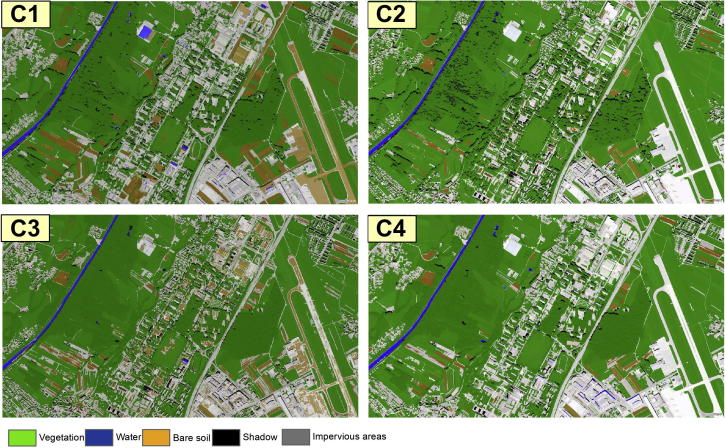
Classification results achieved by the C1–C4 classifications in Test Area 2; C1- expert 1; C2- expert 2; C3- expert 3; C4- classification developed in this paper (author’s classification).

**Fig. 8:**
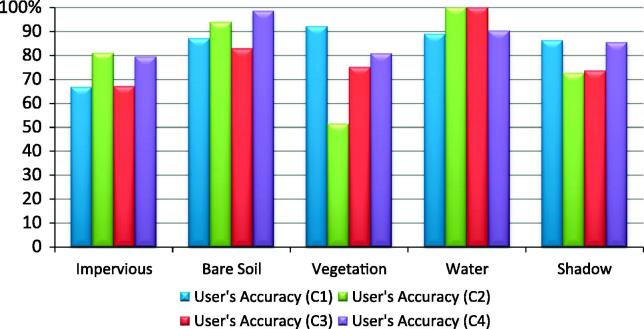
User’s Accuracy per class achieved by C1–C4 classifications in Test Area 2.

**Fig. 9:**
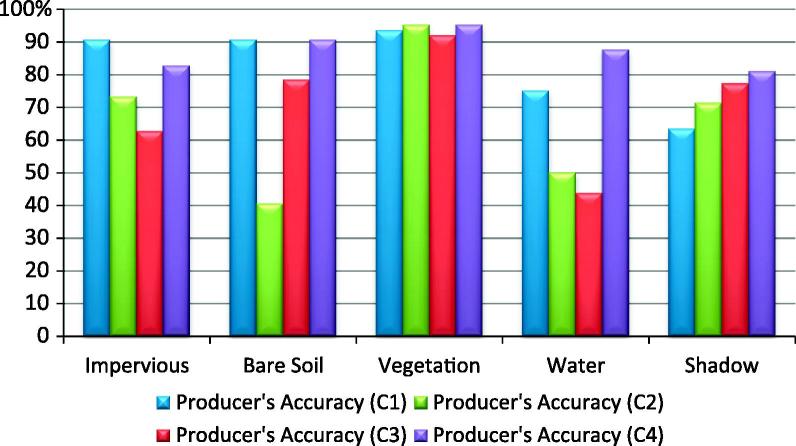
Producer’s Accuracy per class achieved by C1–C4 classifications in Test Area 2.

By applying the C1–C4 rulesets, we obtained the results shown in Fig. 7, Fig. 8, Fig. 9 and depicted in Table 5. By visual inspection, noticeable commission and omission errors of the ‘Impervious Areas’ and ‘Bare Soil’ classes can be observed in Fig. 7, especially in the case of C1 and C3.

**Table 5:** The producer’s and user’s accuracy of the land cover classes achieved by the C1–C4 classifications in Test Area 2; PA%- % producer’s accuracy; UA % – % user’s accuracy; OA – Overall Accuracy (%);kappa (Kappa Index Agreement/Kappa Coefficient); A – Impervious Areas; B – Bare Soil; C- Vegetation; D – Water; E – Shadow.

	C1									C2							
	A	B	C	D	E	Total	PA (%)	UA (%)		A	B	C	D	E	Total	PA (%)	UA (%)
A	68	7	3	1	23	102	90.67	66.67	A	55	7	1	2	3	68	73.3	80.88
B	7	67	0	1	2	77	90.54	87.01	B	3	30	0	0	0	33	40.54	93.75
C	1	0	58	0	4	63	93.55	92.06	C	10	37	59	0	9	115	95.16	51.30
D	2	0	0	24	0	26	75.00	88.89	D	0	0	0	16	0	16	50.00	100.00
E	1	0	1	6	50	58	63.29	86.21	E	11	0	2	14	67	94	84.81	72.048

Total	75	74	62	32	79	322			Total	75	74	62	32	79	326		
							OA	82.29%								OA	70.49%
							kappa	0.78								kappa	0.62
																	
	C3									C4							
A	47	16	2	1	2	68	62.67	67.12	A	62	6	3	2	5	78	82.67	79.49
B	18	58	3	4	0	83	78.38	82.86	B	1	67	0	0	0	68	90.54	98.53
C	4	0	57	0	16	77	91.94	75.00	C	5	1	59	0	8	73	95.16	80.82
D	0	0	0	14	0	14	43.75	100.00	D	1	0	0	28	2	31	87.50	90.32
E	10	0	0	13	61	84	77.22	73.49	E	10	0	0	2	64	76	81.01	85.33

Total	75	74	62	32	79	322			Total	75	74	62	32	79	322		
							OA	73.6%								OA	86.95%
							kappa	0.66								kappa	0.83

The accuracy of the ‘Impervious’ class decreased significantly for the Test Area 2 (Table 5). The C1 achieved the lowest user’s accuracy for the ‘Impervious’ class (66.67%, Fig. 8). The lowest producer’s accuracy for the ‘Impervious’ class was produced by C3 (62.67%). The ‘Bare Soil’ class also reached relatively lower producer’s and user’s accuracy. The C2 achieved the lowest producer’s accuracy (40.54%) because of the overlap with the impervious and vegetation classes (Table 5 and Fig. 9). The confusion with the vegetation is due to the lower values of the NDVI threshold used to mask out the vegetated areas from non-vegetated areas (see the Discussion section for details). C4 achieved an improved user’s accuracy for the ‘Bare soil’ class by in the second test area (98.53%), whereas the producer’s accuracy slightly decreased (90.54%). The accuracy of the ‘Vegetation’ class remained similar, except for C2 which reached the lowest user’s accuracy (51.30 %) because of the confusion with the ‘Bare Soil’ class.

The classification errors of the ‘Water’ class were mainly due to the spectral confusion with the shadowed areas and the industrial buildings with metal roofs. C3 and C2 yielded the lowest producer’s accuracy for the ‘Water’ class (43.75% and 50.00% respectively). The ‘Shadow’ class yielded the lowest producer’s accuracy for C1 (63.29%).

The classification results achieved in Test Area 2 were compared with the results yielded in Test Area1. The results of this comparison are summarized in Table 6 (evaluation based on Eq. (6)). According to the z test, the C2 classification did not perform well in the second test area, whereas C1, C3 and C4 achieved approximately similar results. C1 reached an overall accuracy of 82.29% (about 5 % lower than the accuracy reached in the Test Area 1), whereas the C2 classification achieved an overall accuracy of 70.49% (about 10% lower than the accuracy reached for Test Area 1). The C3 yielded an overall accuracy of 73.6%, (nearly 5 % lower than in the first test area) and the C4 produced an overall accuracy of 86.95% (around 3 % lower than the accuracy achieved in the first test area).

**Table 6:** Summary of the classifications transferability assessment using the *z*-test (Eq. (6)).

		Comparison of overall accuracy		Comparison of proportions	
Classification Test Area 1	Classification Test Area 2	Overall accuracy Test Area 1 (%)	Overall accuracy Test Area 2 (%)	|*z*|	Probability (One tail)
C1	C1	87.36	82.29	1.53	0.126
C2	C2	80.7	70.49	2.909	0.0036
C3	C3	78.24	73.6	1.33	0.183
C4	C4	89.82	86.95	1.09	0.271

## 4 Discussion

We evaluated the variability of the rule-based classification results generated by different operators while performing the same interpretation task. The results of this comparison demonstrated the influence of the analyst’s subjectivity on the classification accuracy. The differences between operators stem mainly from the selection of the classification features, thresholds range settings, and the allocation of the classes of interest to various hierarchical levels. These discrepancies together with the increasing time spent on image classification might impede the application of the OBIA methods in operational frameworks or for large-scale land cover mapping initiatives. This problem was reported in other studies that employed OBIA to derive information from VHR data (Baker et al., 2013, Duro et al., 2012).

C1, C3 and C4 reached consistent results for the second test area, by applying the same classification rules to detect similar classes. These results confirm the findings of previous studies, that rule-based classifications remain consistent to a certain degree when applied to other test areas (Hofmann et al., 2011, Kohli et al., 2013). The moderate decrease of the classification accuracies in the second test area is due to the higher heterogeneity of the new environmental settings. C1 achieved satisfactory results on both test areas. The C2 classification performed well in the first test area, but it proved to be less effective when applied to the second test area. C3 yielded similar results in both test areas, but it also yielded the lowest overall accuracy amongst all classifications. The C4 classification achieved the highest overall accuracy and kappa coefficient for both test areas. This classification relied on the best practices in mapping the land cover classes in urban/suburban areas. These results suggest that well-established libraries of object characteristics might help to define consistent rulesets, and to reduce the time spent on classifying the high resolution imagery.

### 4.1 The shadow class

The C1–C3 classifications defined the ‘Shadow’ class on the first classification hierarchy level (together with vegetation and non-vegetation classes). The C1 and C3 operators used the same feature to classify the shadow class, namely the Yellow band (Band 4), and achieved the lowest producer’s accuracy (for both test areas). While the C1 operator achieved the lowest producer’s accuracy for the ‘shadow’ class (68.75%) in the first test area because this class was misclassified as ‘impervious areas’ class, the lower producer’s accuracy reached by C3 (also 68.75%) was caused by the confusion of the shadow class with vegetation. These differences were generated by the thresholds settings.

At a closer visual examination of the C1 results, we observed that a large number of the shaded objects classified as impervious areas were actually covered by the vegetation class. This misclassification led to an overestimation of the impervious surface. By contrast, the shadows cast by trees on the streets were correctly classified as impervious areas. In this case the reference samples for the shadow class might introduce an error in the classification.

The C2 operator used the ‘mean brightness’ feature to allocate image objects to the shadowed areas. Thus, C2 included both the shadows on vegetation and shadows on the impervious areas in the shadow class. This issue led to the increasing confusion between the shadow class, the dark impervious areas, and the vegetation classes which occurred in both test areas. Again, these results might be influenced by the selection of the reference samples for the shadow class.

The above mentioned problems can be solved by defining two types of shadow classes: shadows on vegetation and shadows on impervious areas. This solution might ease the allocation of the shadows to the proper class: e.g. the shadows on the streets are allocated to the street class if there is a building nearby (Carleer and Wolff, 2006), avoiding the problems introduced by shadow reference samples that are more representative for one or the other shadow class. However, this approach only yields satisfactory results if the shadowed areas consist of the same land cover class. If these areas consist of distinct land cover classes, the ‘shadow’ class must be re-segmented into smaller, semantically homogeneous classes (Verbeeck et al., 2012)

Another source of error associated with the ‘shadow’ class (and subsequent omission and commission errors) might be the misinterpretation of the shadow reference samples, given the difficulty to define the darkness degree of an image object to be classified as shadow (de Pinho et al., 2012). Therefore, the accuracy of the shadow class reported here should be interpreted with caution.

### 4.2 Segmentation issues in OBIA

The image segmentation is an important step in OBIA as it influences to a higher degree the reliability of the classification results (Gao et al., 2011). It might generate over-segmentation (splitting real-world objects into many image objects) or under-segmentation (merging real objects into one image object). The under-segmentation is a “true error” (Liu et al., 2012) that might reduce the classification accuracy dramatically, as the image objects belong to more than one class. In contrast, over-segmented image objects can be further grouped into the classes of interest. For example, the under-segmentation error caused the confusion between ‘vegetation’ and ‘impervious area’ classes occurring in all four classifications. A visual examination of the under-segmented image objects revealed that this segmentation error occurred mainly in areas where the shadows reduced the spectral values of the shaded objects, i.e. the houses surrounded by high trees casting shadows on the nearby houses and streets. In these cases, under-segmentation led to the underestimation of impervious surface area.

The variations of object boundary delineation may affect the degree of agreement between the four classifications. However, three of the operators used the same SPs to identify the classes of interest from the image. Even though the same image objects were used as building-blocks for the further image analysis task, the operators still achieved different results. Therefore, not only the segmentation, but also the ruleset definitions could affect the classifications results.

### 4.3 Image objects feature selection and threshold definitions

The rulesets were different among the operators except for the rulesets used to classify the vegetation areas, and the rulesets used by C1 and C3 to identify shadowed areas. As proven by our experiment, using the same features to discriminate between different land cover classes does not guarantee similar classification results. The above discussion about the shadow rulesets defined by C1 and C3 supports this conclusion. Another example is the definition of the NDVI threshold leading to the misclassification of bare soil as vegetation. The lower the NDVI threshold, the more image objects are assigned to the vegetation class. Previous research has shown the difficulty of identifying the proper NDVI threshold to separate less dense vegetation area from bare soil areas (de Pinho et al., 2012). The semantics of the class to be identified in the image also plays an important role in determining the adequate threshold for the feature selected to classify the class. For example, Corcoran et al. (2010) has proven the influence of the operators’ conceptualization on the accuracy of the real world objects delineation. Thus, different conceptualizations of the real world geographic objects might influence the definition of the thresholds for the features selected to identify the classes of interest. To avoid this issue, we provided all participants in the experiment with class definitions as precise as possible. An example to illustrate such definition is the bare soil class, defined as “Areas with no dominant vegetation cover. 50% of the ground or more is bare”.

As an inappropriate threshold selection might lead to the misclassification of the desired land cover classes and inconsistencies of the developed classification rulesets, we need solutions to identify the proper threshold intervals for the selected object features. For example, the optimal NDVI threshold can be identified using reference data (Pu and Landry, 2012), or by using algorithms designed for the automatic extraction of the thresholds (Martha et al., 2011, Sebari and He, 2013).

The inclusion of texture information and the Red-Edge/Green index seem to be suitable for classifying bare soil areas. The GLCM Homogeneity texture on the Red Band worked well because the soil is more porous and rougher compared to the buildings with bright-red roofs. Previous studies proved the suitability of the texture parameter for land cover classifications (Carleer and Wolff, 2006, Kim et al., 2011, Moller-Jensen, 1997) and its stability when applied to other test areas (Kohli et al., 2013). The RedEdge/Green Index was introduced to discriminate barren areas from other impervious surfaces. It does not perform well to separate the soil class from the buildings with the green roof.

## 5 Conclusion

In this paper, an experiment was conducted to evaluate the differences between rule-based classifications implemented by different experts assigned the same image analysis tasks. The experiment was carried out using WV2 imagery. The magnitude of differences was quantified with kappa statistics, and statistical significance of the differences between pair-wise classifications was evaluated using the McNemar’s test. All classifications turned out different because of: (i) the features used in the developed rules to determine whether the image objects belong to a specific class or not; (ii) the definition of the threshold intervals for the selected features; (iii) the allocation of classes to the designed hierarchical classification levels. These differences influenced the overall classification accuracy. The transferability assessment proved that rule-based classifications remain consistent when applied to an additional test area (using the same satellite imagery type).
